# What impact will the achievement of the current World Health Organisation targets for anthelmintic treatment coverage in children have on the intensity of soil transmitted helminth infections?

**DOI:** 10.1186/s13071-015-1135-4

**Published:** 2015-10-22

**Authors:** JE Truscott, HC Turner, RM Anderson

**Affiliations:** London Centre for Neglected Tropical Disease Research, London, UK; Department of Infectious Disease Epidemiology, School of Public Health, Faculty of Medicine, St Mary’s Campus, Imperial College London, Norfolk Place, London, W2 1PG, , UK

## Abstract

**Background:**

It is the aim of the World Health Organisation to eliminate soil-transmitted helminths (STH) as a health problem in children. To this end, the goal is to increase anthelmintic treatment coverage for soil transmitted helminths to reach 75 % in pre-school aged and school aged children by 2020 in endemic countries. In this paper, we use mathematical models to investigate the impact of achieving this goal on the burdens of *Ascaris lumbricoides*, *Trichuris trichuria* and hookworm.

**Methods:**

We employ a deterministic fully age-structured model of STH transmission and mass drug administration to examine the changes in worm burden in response to the known and projected coverage trends in children up to 2020 and beyond. Parameters are estimated from worm expulsion data and age intensity profiles before treatment using maximum likelihood methods. Model validation is performed using reinfection studies for *Ascaris* and analyses are conducted to assess the sensitivity of the predicted outcomes to variation in parameter estimates including transmission intensity (R_0_), children’s contributions to the pool of infective stages and drug coverage levels.

**Results:**

The impact of the required increase in coverage trends are quite different across the three species. *Ascaris* burdens are reduced dramatically by 2020 with elimination predicted within studied the setting a further 10 years. For *Trichuris* and hookworm, however, impact is more limited, due to issues of drug efficacy (*Trichuris*) and distribution of worms in the population (hookworm). Sensitivity analysis indicates that results are largely robust. However, validation against *Ascaris* data indicates that assumptions concerning re-infection among children may have to be revised.

**Conclusions:**

The 2020 coverage target is predicted to have a major impact on *Ascaris* levels by 2020. However, there is evidence from model validation that *Ascaris* in children is more resilient to treatment than currently assumed in the model. Broader coverage across all age classes is required to break transmission for hookworm and alternative dual drug treatment approaches are needed for *Trichuris*.

**Electronic supplementary material:**

The online version of this article (doi:10.1186/s13071-015-1135-4) contains supplementary material, which is available to authorized users.

## Background

The World Health Organization’s (WHO) policy for control of the soil transmitted helminths (STH) largely centres on two groups, preschool aged children (pre-SAC), and school-aged children (SAC). The strategy for treatment is based on the argument that pre-SAC and SAC often harbour heavy infection which will have a detrimental impact on anaemia, child growth, and development. Although this largely holds for *Ascaris lumbricoides* and *Trichuris trichuria,* where the intensity of infection is highest in these age groupings, it holds less well for the hookworm species since infection is often greatest in adults [1]. The current WHO guidelines focus on SAC, both for monitoring infection and as a target for treatment, although treatment of pre-SAC and women of childbearing age is also recommended where sustainable delivery mechanisms exist, especially in areas of intense transmission [2, 3]. The guidelines recommend treating SAC annually where any STH prevalence falls between 20 % and 50 % and twice a year where it exceeds 50 % [2].

**Fig. 1:**
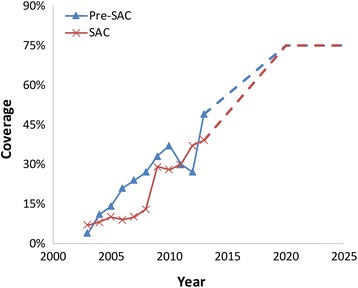
Average national percentage coverage of Pre-SAC (blue line) and SAC (orange line) with STH drug treatment in areas of endemic infection as reported by WHO [13] (solid lines). Linear extrapolation is used for the interval 2013 – 2020, after which coverage is assumed to remain at 75 % (broken lines). Drug efficacy is as defined in Table 1

WHO aims to scale up mass drug administration (MDA) for STH, so that by 2020, 75 % of the pre-SAC and SAC in need will be treated regularly [3–6]. Progress has been good in some areas, but less so in others (Fig. 1). Pharmaceutical companies who manufacture the main anthelmintics have continued to increase drug donations. Slow progress in raising coverage in some countries is due in part to the logistical challenges in getting donated drugs to rural populations, who are often beyond “the end of the road.” At present, many countries with endemic STH infections are not availing themselves of the freely donated drugs to treat children.

The current situation with coverage as reported by WHO is summarised in the third progress report of the London Declaration [5]. In 2013, global coverage of those in need was 39 % for SAC and 49 % for pre-SAC [6]. In 2015, STH moved from yellow to green in the progress score card chart (recently developed by Uniting to Combat NTDs), in part due to better coordination between UNICEF and WHO which has led to an improvement in reporting of coverage for pre-SAC. The coverage in this age grouping now exceeds 50 %. Coverage in SAC is reported as 39 %, which, it is argued in the report, is on track for a 75 % target in 2020.

We present the results of our analysis of the predicted impact of achieving 75 % MDA coverage in pre-SAC and SAC by 2020 on the intensity of infection as a function of host age. The study was conducted under the umbrella of the Neglected Tropical Disease Modelling Consortium, which is supported by the Bill and Melinda Gates Foundation [7]. To reflect different settings, where one or other of the three major STH species may be the dominant infection, analyses are performed separately for *Ascaris*, *Trichuris* and the hookworms. The calculations are based on deterministic mathematical models of the transmission dynamics of the parasites under MDA with key parameter estimates derived from field worm expulsion epidemiological studies [8, 9]. The analysis is based on the premise that the WHO MDA coverage targets are reached by 2020. We also assess the time taken once reached for the transmission dynamics to settle to its new steady state, whether this be the persistence of infection at a lower level than prior to scaling up MDA or elimination, if the breakpoint in transmission is breached in, for example, low or medium transmission settings.

## Methods

A deterministic fully age-structured mathematical model of the transmission dynamics of helminths with a direct lifecycle is used to examine the impact of MDA on the pre-SAC, SAC and adult age groupings. The structure of this model has been described in a series of recent publications [10, 11] and in the Additional file 1 to this paper. It is based on a model framework first described by Anderson & May [12]. The model describes the evolution of the mean worm burden in a host as a continuous function of age, although results are generally presented as averages across the pre-SAC, SAC and adult age groups. Hosts contribute infectious material to a single reservoir and are re-infected from it. The model is based on a negative binomial distribution for worms in a host and hence the probability of a host of a given age having a given worm burden can be directly calculated from the output. Stochastic individual based simulations have also been performed but are not reported in this paper since the mean intensity of a large number of runs is close to the deterministic results. Stochastic simulations permit the examination of the impact of predisposition to infection and variation in adherence to treatment at each round of treatment but these results will be reported in a separate paper.

Treatment coverage in the pre-SAC and SAC age groupings (2–4 years of age and 5–14 years of age respectively) was represented by the national percentage coverage of pre-SAC and SAC with STH drug treatment in areas of endemic infection as reported by WHO [13], shown in Fig. 1. A figure of 75 % was assumed for both age groupings in 2020 and beyond and coverage for the years 2013 to 2020 was determined by linear interpolation. These values represent national means and coverage in individual locations will vary considerably from these values. However, given sufficient linearity in the response of the model to variation in coverage, results should be indicative of large-scale national trends in worm burden. The drug efficacies for albendazole for the three species were those reported by Vercruysse et al. [14] and listed in Table 1.

**Table 1:** Parameter values estimated using maximum likelihood methods and used in the numerical evaluations of model predictions for each parasite

Parameter	*Ascaris*	*Trichuris*	Hookworm	Source
Basic Reproductive number, R_0_	2.12	1.72	2.34	Fitted
Adult worm life expectancy	1 year	1 year	2 years	[12]
Negative binomial clumping parameter, k	0.90	0.38	0.35	Fitted
Density dependence fecundity parameter, ɣ	0.07	0.0035	0.08	Fitted
Age-specific transmission parameter, β_i_ for age group i’s contact with infectious reservoir. Relative egg contribution by age, ρ_i_, proportional to β_i_.	0.22, 1.88, 1, 0.53	0.5, 2.13, 1, 0.28	0.03, 0.09, 1, 2.5	Fitted
Drug efficacy as a proportion of worms killed by Albendazole	0.99	0.50	0.95	[14]
Source of age - mean intensity profile for fitting	Elkins et al. South India [15]	Bundy et al. St Lucia [20]	Bradley et al. Zimbabwe [17]	-

The age intensity profiles from the cited sources based on worm expulsion were used to derive estimate of R_0_, β_i_ and k. The values of ɣ were derived from egg per gram of faeces and worm expulsion counts [transmission age groups: Ascaris and hookworm: 0–2;2–5;5–15;15+; Trichuris: 0–2;2–7;7–12;12+]

**Fig. 2:**
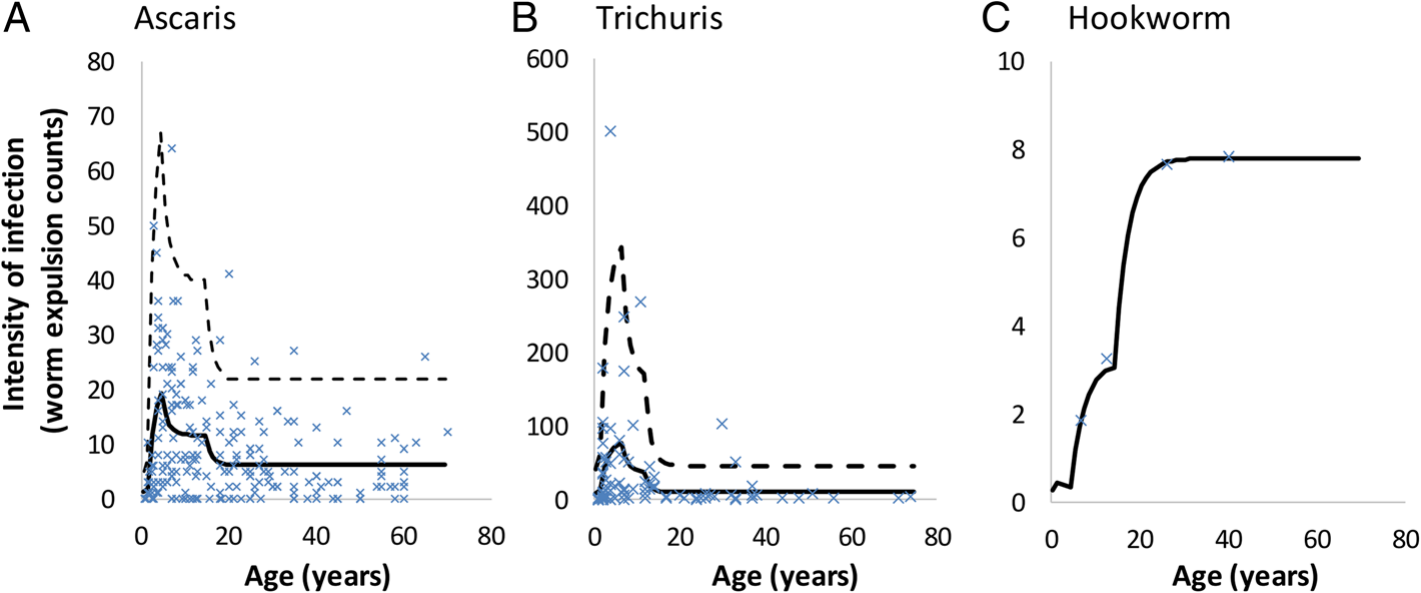
Maximum likelihood estimator fits to worm expulsion data for *Ascaris* (Panel **a** data from Elkins et al.), *Trichuris* (Panel **b** data from Bundy et al.), and Hookworm (Panel **c** data from Bradley et al. – *Necator americanus*). The fits are performed for individual worm counts for *Ascaris* and *Trichuris*, and for mean counts for hookworm (in the absence of individual-level data)., The solid line is the mean individual worm burden as a function of age for the maximum likelihood parameter estimates and the dotted line is the 97.5 percentile for individual worm burden from the model, based on the assumed negative binomial distribution. The crosses are the raw data

Parameter assignments for the major population and transmission processes were as defined in Table 1 based on epidemiological studies of the three major STH species, involving cross-sectional worm expulsion studies across the full population age range. Parameters were fitted to age-expulsion worm count paired data for the three major STH species using maximum likelihood techniques. Additional information was obtained from paired worm burden/egg output data, where possible from the same study. The methods used are discussed in the SI. The worm expulsion profiles chosen for the three STH species were as follows: *Ascaris lumbricoides* in India – Elkins et al. [15]; *Trichuris trichuria* in St Lucia – Bundy et al. [16]; hookworm (*Necator americanus*) in Zimbawe – Bradley et al. [17] (Fig. 2). Where possible, we have estimated parameters using the full age structured model and all relevant data. As a result, parameter values may differ from those quoted in the original studies, where parameters are usually estimated separately.

Treatment coverage is assumed to be at random at each round of MDA in each age grouping. This is unlikely to be true in reality and a future publication will examine compliance issues within a stochastic framework. If those not treated are predisposed to this state for social or behavioural factors, the impact of MDA at 75 % coverage will be less than predicted in the results discussed in this paper.

Host demographic details (age dependent death rates and population pyramids) were taken from data published  by the DHS program with the profile in Kenya in 2003 chosen to represent a typical profile [18].

### Model validation

Validation of model predictions is essential and can be performed in a variety of ways. Qualitative comparisons of model prediction with observed pattern of reinfection post mass treatment is one approach. Ideally, however, quantitative comparisons of predicted outcome of the impact of MDA require parameter estimation using data from a defined population with little or no past treatment and then the comparison of expected and observed patterns of reinfection after intervening rounds of chemotherapy. Precise information on who has been treated and how often is very valuable in performing such comparisons, but rare in practice. In comparing model predictions with observations, the quality of agreement can only be judged in the light of the uncertainties associated with the model. The current model describes the evolution of the negative binomial distributions that describe worm burdens at a given age and so readily lends itself to the construction of credible intervals for the observed data. Beyond this, however, there are known biases in common diagnostic techniques, with false negative results leading to underestimates in intensity and prevalence measures. These effects are as yet poorly characterised and not included in our model. New tools, such as quantitative PCR, offer some hope for the future in providing precise scores for prevalence and perhaps even egg burdens in stools. A further complication arises from density dependence in egg production by female worms which can result in higher per capita egg output post the initial round of treatment during the early phases of reinfection as recorded by Elkins et al. [19].

Model validation has rarely been attempted for helminth infections, but it is a central need for ongoing and future epidemiological studies of MDA impact, if predictions on coverage requirements to interrupt transmission are to carry weight amongst public health policy makers.

We report a validation exercise using a reinfection study of *Ascaris* recording prevalence, epg, epd (eggs per female worm per day) and worm expulsion in a community in southern India that had not previously been treated prior to the initiation of mass drug administration [15, 19]. We also examine the sensitivity of model predictions on the impact of achieving 75 % coverage in pre-SAC and SAC to the value of basic reproductive number (R_0_) which records the transmission intensity in a defined location.

## Results

**Fig. 3:**
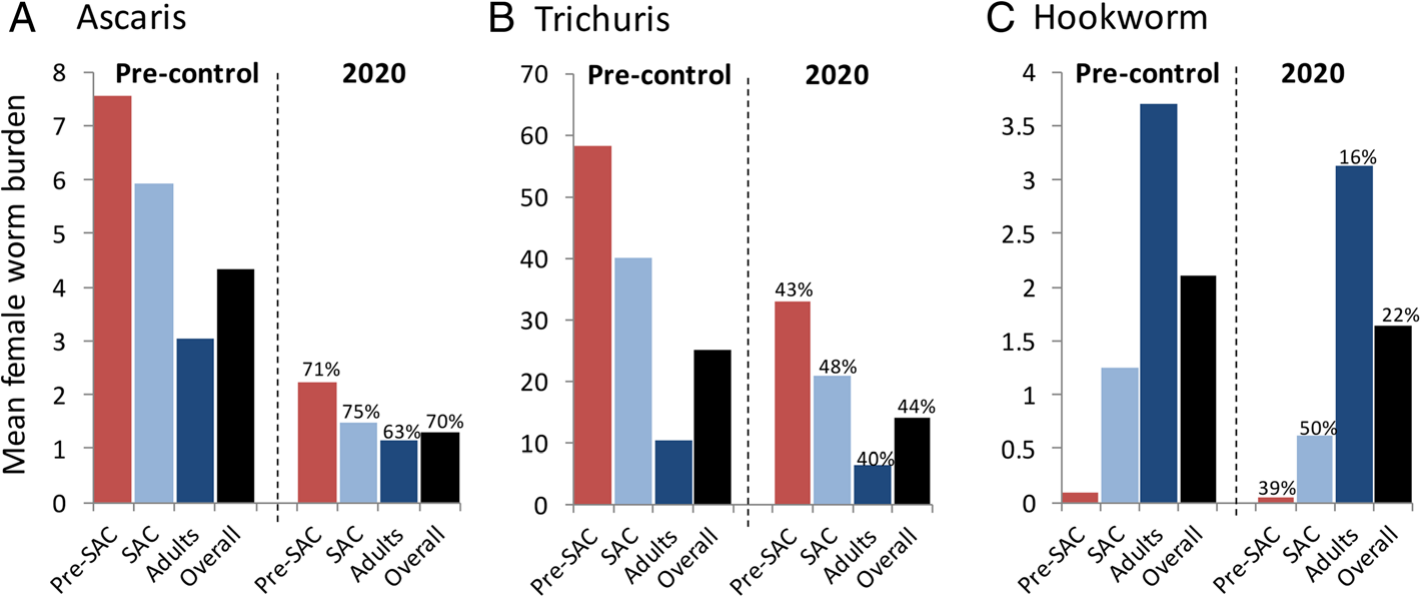
Predicted *Ascaris* (panel **a**), *Trichuris* (panel **b**) and hookworm (panel **c**) burdens in Pre-SAC, SAC, Adults and the overall population mean prior to treatment and in 2020 with 75 % treatment coverage of Pre-SAC and SAC. No adult coverage is assumed. Percentages indicate the respective worm burden reduction. Parameter values are as defined in Table 1

We present the results of the numerical evaluations of model behaviour in the form of a set of graphs for each of the three main STH species in settings where MCMC methods were employed to estimate R_0_ and the egg production and contact parameters for three age groupings, pre-SAC, SAC and Adults (Fig. 3). The transmission parameter, R_0_, defines the average number of off spring produced by one female worm that transmit and survive to reach reproductive maturity in the human host (7). The values chosen for the three parasites are as listed in Table 1.

The first set of graphs are histograms recording the initial mean endemic female worm burden in pre-SAC, SAC and Adults (15+ years old) and the respective worm burdens at 2020 with the prescribed treatment coverage levels in 2020 with a time series of coverage as noted in the Methods section and Fig. 1. The results for the three species of STH are recorded in Fig. 3 for *Ascaris*, *Trichuris* and hookworm.

The impact of achieving the goals of 75 % coverage of Pre-SAC and SAC was different for the different STH species and for various age groupings. The predicted impact for *Ascaris* is much greater than that for hookworm and *Trichuris*. The impact on *Trichuris* was similar to *Ascaris*, but lower due to the lower drug efficacy (see Table 1). It should be noted that with the assumed age profile (derived from a set of field studies in St Lucia [20]) from which parameters were estimated there was a low pre-control worm burden in the adults (who are untreated). In settings where adults have a higher pre-control burden the impact would be even less.

The impact on hookworm was lower for all three age groups, compared to *Ascaris* and *Trichuris*. The overall worm burden was only reduced by 22 % because the burden of hookworm infection lies largely in untreated adults due to the rising burden of infection with age (Fig. 2) This effect is also observed in other models of PCT for hookworm [21]. Consequently, child-targeted treatment does not significantly impact the overall level of transmission in the community. Children are therefore likely to become reinfected quickly after treatment from the adult reservoirs of infection [22].

**Fig. 4:**
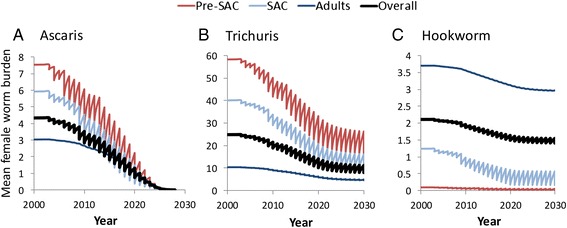
Changes over time from 2000 to 2030 in a medium transmission setting (R_0_ values are around 2.0 – see Table 1) of the average female *Ascaris* (panel **a**), *Trichuris* (panel **b**), and hookworm (panel **c**) burdens in Pre-SAC, SAC and adults. Treatment trend as described in Fig. 1. The overall population-average worm burden is plotted as a solid black line

Further assessment of model predictions is provided in Fig. 4 which plots changes in mean female worm burden for the three STH species form the start of treatment to 2020 where 75 % coverage of both Pre-SAC and SAC is achieved and for a further 10 years at the 2020 levels. The plots record predicted changes in worm burdens in Pre-SAC, SAC and Adults. The overall population mean is also plotted as a solid black line. The projections show that although projected coverage levels stabilise after 2020, the full impact on worm burden is generally not achieved until some years later. For Ascaris, the final coverage level is just sufficient to eliminate the parasite in the studied setting, but that is not achieved for a further 5–6 years. Likewise, Trichuris does not reach its new equilibrium for a similar length of time. Hookworm stabilises much more rapidly, since treatment has had a relatively small effect on the overall worm burden in the population.

Note that the total benefit of achieving the current coverage goals is dependent on the rate that treatment coverage is scaled up from 2015 until 2020. The faster the rate of scale up, the greater the total impact of MDA.

**Fig. 5:**
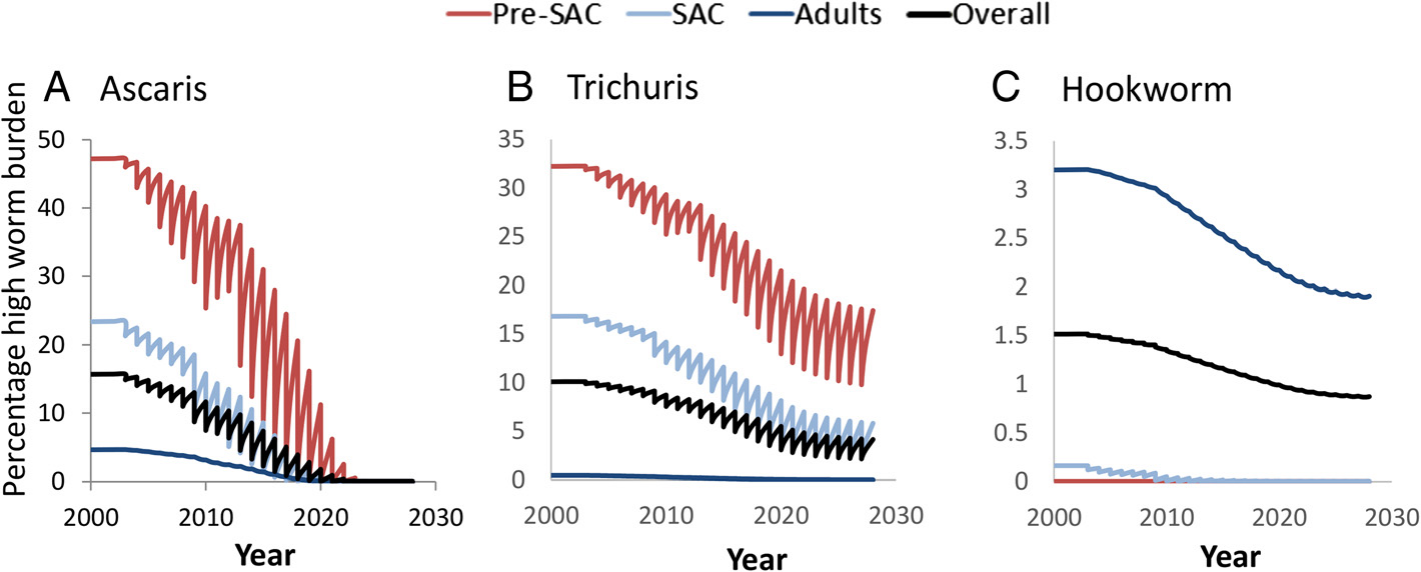
Evolution of the percentage individuals with worm burdens above morbidity thresholds (see main text) for *Ascaris* (panel **a**), *Trichuris* (panel **b**), and hookworm (panel **c**) in Pre-SAC, SAC, adults and also over the whole population, with treatment trend as shown in Fig 1. Parameterization as for simulations in Fig. 4

Figure 5 shows the equivalent trend in the percentage of individuals with worm burdens above a threshold associated with morbidity and developmental deficits [23]. The thresholds are defined for each of the STH species and are categorised into 3 age categories: [0–4 years], [5–9 years], [10+ years]. These thresholds have been used to define morbidity and disease burden and the impact of treatment in a range of studies [24–27]. The effect of increasing coverage on high worm burden is broadly the same as the effect on mean worm burden (Fig. 3), but changes in mean worm burden having a smaller impact on high burden percentage when mean worm burden is high and a larger impact when mean worm burdens are low. As a result, the impact on high worm burden in SAC and pre-SAC is very strong for hookworm, but this hides the fact that relatively high mean worm burdens still exist in adults and hence in the population as a whole. As with all indirect measures of disease burden, the high burden thresholds are subject to considerable uncertainty. The effect of this is addressed in the Additional file 1.

### Sensitivity analyses

The predictions presented in Figs. 3, 4 and 5 are based on MLE parameter estimates derived from particular baseline datasets, parameters taken from the literature and predictions of future trends in coverage that will meet WHO goals. However, different baseline datasets will yield different parameter values and the estimation process itself introduces uncertainty in parameter values. Equally, the 75 % target coverage will vary on a local level, with some communities achieving higher rates and others falling short. In Table 2, we examine the impact of such uncertainties in key parameters on the percentage drop in the predicted outcomes by 2020. The table records the variation in predicted outcomes for changes in key parameter values above and below the value estimated from data. The parameters examined are the basic reproductive number (R_0_ = +1.0, −0.5), MDA coverage at 2020 (60 % or 90 %) and the age-specific transmission parameter β_i_ for the Pre-SAC and SAC age groups (+ or – 50 %, to reflect the importance of children in contributing to the pool of infective stages for the whole population). As already observed, the impact of assumed coverage trends on Ascaris is sufficient to eventually bring about elimination in the studied setting, while for Trichuris and hookworm, species-specific factors limit its effect (low drug efficacy for the former and most worms being in adults for the latter). The degree of impact is reflected in the sensitivity, with Ascaris generally highly sensitive to changes and the other two species much less so. This further highlights the need to consider alternative strategies for MDA for *Trichuris* and hookworm: the fact that quantitative change has little effect on outcome points to the need new approaches to control. Other control measures such as latrines in schools, the wearing of shoes (in areas where hookworm is the dominant STH) and improved hygiene in general, can, in combination with MDA, may be necessary to achieve long-term goals. Looking in more detail, it’s clear that Ascaris and Trichuris are much more sensitive to transmission intensity variations than hookworm. This is a consequence of the dominant role of children in the reinfection cycle in the former species as opposed to hookworm, where adults drive reinfection. Perhaps most surprising is the relative lack of sensitivity of the outcome to changes in the exposure of children to infectious material. The variation examined represents a factor of 3 between highest and lowest value, but the outcome only varies by about 10–15 %. The relative values of the exposure parameter are determined by the details of the baseline age profile shape, which can vary significantly between studies (e.g. for hookworm [1]). It is reassuring from the modelling point of view that results of interest are not overly sensitive to potentially variable aspects of the underlying data.

**Table 2:** Sensitivity analyses of model projections to changes in the values of key parameters

	Pre-SAC	SAC	Adults	Overall population
Ascaris				
Fitted parameters (see Table 1)	71	75	63	70
Higher and lower transmission setting (R_0_ + 1.0, −0.5)	35, 99	45, 99	24, 99	36, 99
Coverage level achieved in 2020 (60 %, 90 %)	59, 82	64, 85	51, 73	58,80
The degree of exposure and contribution of Pre-SAC and SAC to the infectious reservoir (β_2_ and β_3_ + 50 %, −50 %)	83, 48	85, 56	76, 37	82, 45
Trichuris				
Fitted parameters (see Table 1)	43	48	40	44
Higher and lower transmission setting (R_0_ + 1.0, −0.5)	24, 80	31, 81	20, 77	24, 79
Coverage level achieved in 2020 (60 %, 90 %)	37, 49	42, 54	34, 45	38, 49
The degree of exposure and contribution of Pre-SAC and SAC to the infectious reservoir (β_2_ and β_3_ + 50 %, −50 %)	45, 38	50, 43	43, 32	46, 37
Hookworm				
Fitted parameters (see Table 1)	39	50	16	22
Higher and lower transmission setting (R_0_ + 1.0, −0.5)	36, 43	48, 54	12, 21	19, 27
Coverage level achieved in 2020 (60 %, 90 %)	34, 43	45, 55	14, 17	20, 24
The degree of exposure and contribution of Pre-SAC and SAC to the infectious reservoir (β_2_ and β_3_ + 50 %, −50 %)	45, 33	55, 45	24, 6	32, 10

The numerical values record the percentage reduction in the predicted mean intensity of infection in each age group from a rising treatment coverage to 75 % in 2020 as compared to baseline in 2003. The default parameters are in Table 1

### Interrupting transmission

Breaking transmission is not yet the aim of current WHO policy, which is currently targeted at reducing morbidity in children. However, there is growing interest in investigating the feasibility of interrupting transmission of STH by crossing an unstable breakpoint in the parasite’s transmission dynamics created by the dioecious nature of sexual reproduction which requires the presence of female and male worms in the same host to produce viable offspring [28–30].

Previous work has shown that achieving the current coverage goal could lead to the elimination *Ascaris* in certain transmission settings with low R_0_ values (around 2) as detailed in a series of recent publications [10, 11, 31], although this outcome is particularly sensitive to the value of the fecundity parameter, *γ*. The current Ascaris parameter regime falls within this range, as can be seen from Fig. 4. For higher R_0_ values (R_0_ = >2.5), transmission settings, models predict that elimination can only be achieved by extending treatment to adults to some degree or by increasing the frequency of treatment to twice a year [11].

The current target of 75 % coverage of Pre-SAC and SAC was not projected to be sufficient for the elimination of *Trichuris* and hookworm (as indicated in Fig 4). This is because the majority of hookworms are harboured by adults, and for *Trichuris* the treatment efficacy of albendazole and mebendazole monotherapy is low [14, 22, 32]. Consequently, the elimination of hookworm requires the treatment of adults, and the elimination of *Trichuris* requires the use of a two drug treatment combination that has a higher drug efficacy (such as ivermectin co-administration with albendazole or mebendazole) [H C Turner et al.,‘An economic evaluation of expanding hookworm control strategies to target the whole community’, submitted; H C Turner et al., ‘Analysis of the population-level impact of co-administering ivermectin with albendazole or mebendazole for the control and elimination of Trichuris trichiura’, submitted].

Table 3 records the minimum number of annual rounds of treatment required for *Ascaris* to be eliminated (under the current parameters) as a function of proportional treatment coverage in Pre-SAC and SAC. In this case, the trend in coverage is not included and it is assumed that treatment is maintained at the given coverage level throughout. It is important to note that these are deterministic projections. Stochastic simulations show that to achieve success with a high degree of certainty, longer durations of high coverage are required. Table 3 indicates that elimination is still possible at a range of coverage levels less demanding than 75 %, albeit at a greater number of years of treatment. The number of rounds quoted in Table 3 is from the untreated baseline. Given the impact of the trend up to 2020, the number of additional years will be less than those quoted in the table. The symmetry of Table 3 shows that the contribution of SAC and Pre-SAC groups towards elimination is approximately equal under the current parameter regime. While the Pre-SAC age group is less accessible to treatment than SAC, it also represents a smaller fraction of the population. These factors may prove important as WHO goals switch from control to elimination in future.

**Table 3:** The number of rounds of annual treatment (hence years) in Pre-SAC and SAC required to eliminate transmission for *Ascaris* as a function of coverage in Pre-SAC and SAC (parameter values as defined in Table 1)

	Coverage of SAC											
		0	10 %	20 %	30 %	40 %	50 %	60 %	70 %	80 %	90 %	100 %
Coverage of Pre-SAC	0	NA	NA	NA	NA	NA	NA	NA	NA	NA	NA	NA
	10 %	NA	NA	NA	NA	NA	NA	NA	NA	NA	NA	23
	20 %	NA	NA	NA	NA	NA	NA	NA	NA	NA	21	16
	30 %	NA	NA	NA	NA	NA	NA	NA	NA	20	15	12
	40 %	NA	NA	NA	NA	NA	NA	NA	20	15	12	10
	50 %	NA	NA	NA	NA	NA	NA	21	15	12	10	8
	60 %	NA	NA	NA	NA	NA	22	15	12	10	8	7
	70 %	NA	NA	NA	NA	24	16	12	10	8	7	6
	80 %	NA	NA	NA	NA	17	12	10	8	7	6	6
	90 %	NA	NA	NA	18	13	10	8	7	6	6	5
	100 %	NA	NA	21	14	11	9	7	6	6	5	5

NA denotes that it is not possible to achieve elimination within 25 years with the defined level of coverage in Pre-SAC and SAC

### Model validation – Ascaris

As outlined in the Methods section, validation of model predictions requires comparison of predictions post treatment with observed outcome based on information on parameter estimates and initial conditions and data on who is treated and how frequently and who is surveyed. Given the nature of the models, the gold standard data is individual and longitudinal with expulsion data at the initial and final surveys and a history of treatment. Few published epidemiological studies on STH match this level of rigour. One such study that meets the criteria of worm counts at two time points, plus information on demography for the village treated and details of MDA coverage, is that by Elkins et al. [15, 19]. We are in the fortunate position of having access to the raw data for each individual treated for this study, despite the fact that is was carried out in 1983–84. This raises an issue concerning STH epidemiological studies. Far too few record data electronically and make such data freely available electronically. This is an important need in the coming years as MDA coverage is hopefully increased. The arm of the study providing the current data comprises a cross sectional survey using expulsion techniques covering 68 % of the population in January 1984, followed by a re-infection survey in December of the same year, also providing worm burden data.

**Fig. 6:**
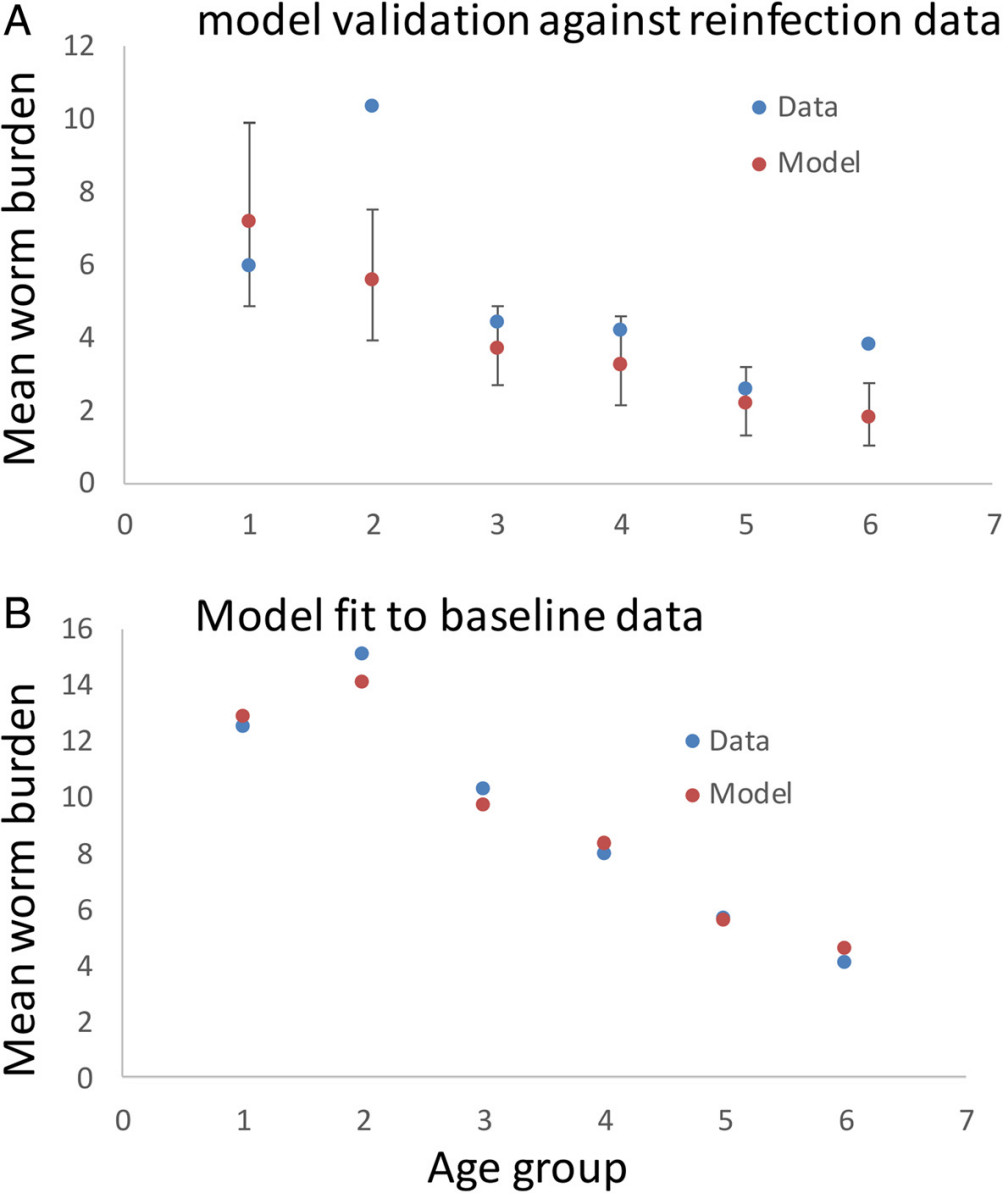
Validation of model behaviour. Panel **a** records the mean worm burden and 95 % credible intervals for the predicted mean intensity of infection by age class (1 = 0–5 years; 2 = 5–10 years; 3 = 10–20 years; 4 = 20–30 years; 5 = 30-45years and 6 = 45–70 years), plotted against the observed mean values recoded in the raw data from the Elkins et al. study of *Ascaris* reinfection [19]. Graph **b** records the original baseline data as mean intensity of infection by age group and the model fit carried out to estimate the key parameters (k, γ, β_i_, and R_0_). T

The results of the validation analysis on the Elkins et al. data is displayed in Fig. 6. Panel a records the fit of model predictions and their 95 % credible intervals, to the observed mean worm counts for each age group after 11 months of reinfection. Graph b records the original model fits to the baseline data from which parameter estimates were initially derived to make the reinfection predictions using maximum likelihood. Note that no revised parameter estimates were made using the reinfection data – so this is ‘true’ validation, in the sense of using the predefined model to make predictions of MDA impact. Note the fit is good overall, but less good for the 5–10 year old age group. The reasons for this mismatch are unclear at present. A variety of factors could be important, such as much higher recycling of infection within this age group (they perhaps largely infect themselves), or changes in worm aggregation between age classes. We assumed an overall k value for the negative binomial distribution of worms per person in the total population. We know that k varies with age [33, 34], and this could influence the quality of the fit. Both issues will be explored in future work.

## Discussion

We summarise the main conclusions of our analyses by STH species, before turning to more general considerations.

The stated aim of WHO to achieve 75 % coverage by 2020 for children will have a very considerable impact on *Ascaris*. In areas of low and medium transmission (R_0_ < 2.5) such coverage will eventually result in transmission interruption given it is sustained or treatment frequency is altered to biannually. In areas of high transmission with limited past experience of treatment, high coverage levels are required or coverage should be extended to include adults.

For *Trichuris*, the situation is less encouraging given the poor efficacy of albendazole and mebendazole for this parasite. As discussed elsewhere [H C Turner et al., ‘Analysis of the population-level impact of co-administering ivermectin with albendazole or mebendazole for the control and elimination of Trichuris trichiura’, submitted], dual drug therapy involving ivermectin co-administration with albendazole or mebendazole is required to achieve very low burdens by 2020. Current treatment guidelines will not achieve a great deal and hence a revision is required for areas where *Trichuris* is the dominant STH.

A similar picture emerges for hookworm, but for different reasons. As discussed in previous publications [22], most worms are harboured by adults and hence treatment coverage must be broaden to encompass this age group.

In all three cases some ‘herd’ impact on transmission can be observed in adults as a consequence of treating Pre-SAC and SAC (Figs. 3 and 4). Such effects have been observed in practice in, for example, areas of high *Trichuris* transmission [20]. In all cases, however, the speed of gearing up coverage to 75 % will be crucial. Trying to achieve this level before 2020 is highly desirable. The overall impact for all could also be improved by extending current guidelines to encompass adults wherever possible, perhaps by using the school as a focus for treatment [H C Turner et al., ‘An economic evaluation of expanding hookworm control strategies to target the whole community’, submitted].

The coverage trend shown in Fig. 1 which drives the evolving parasite burdens represents an average coverage across many countries. In reality, this will hide considerable geographical variation. Our sensitivity analysis looked at the response to the coverage level achieved in 2020, which partially addresses this question. The indication is that, for Ascaris, large variations in coverage across countries will result will result in areas of elimination interspersed among areas in which the parasite is still endemic. For Trichuris and hookworm, the resultant variation is likely to be much less. On a national scale, the linearity in the response of the models to variation in coverage (See Table 2) indicates that average impact will be well represented by average coverage.

Looking at the percentage of high burden individuals in the population, the trend under increasing coverage broadly matches that seen for the mean worm burden in different age categories and overall, with the distinction that the impact on the prevalence of high burden individuals is much greater when mean burden is low. Perhaps more important is the sensitivity of high burden prevalence to the threshold values that define it, as discussed in the Additional file 1. The range of uncertainty in these values carries across into large changes in the absolute values of high-burden prevalence in age groups and in the ratios between them.

The WHO guidelines state that one of the goals of preventive chemotherapy (PCT) is the reduction of heavy burden in children (SAC and pre-SAC) to less than 1 %, as defined by moderate to heavy egg output [35, 36]. While egg count-based metrics are easier to apply to collected data, as a proxy for high worm burden, they have important drawbacks. The connection between worm burden and prevalence of moderate to high egg count can only be made with the knowledge of the net egg output per worm and variability in egg output for a given worm population. These parameter values are either largely unknown (variability) or exhibit a large range across different studies (egg output per worm [37]). In addition, evidence suggests that the distribution of egg output per worm has a high variance and skew [38, 39], resulting in potentially inaccurate and biased assessments of the underlying worm burden. As such, this suggests that moderate to high egg counts are a poor quality indicator of underlying worm distribution.

More generally, we have explored sensitivities to variation in key parameter estimates but in qualitative terms the above conclusions hold. Other factors, however, could influence the validity of these predictions.

The first concerns acquired immunity dependent on past exposure to infection. At present the evidence for this as a key factor in the acquisition of infection is limited, despite the many host responses to parasite antigens. If a degree of acquired immunity was important in limiting the acquisition of infection, especially in adults, then our conclusions will be too pessimistic in the short term (post treatment reinfection restricted by past exposure) but too optimistic in the longer term once repeated treatment has acted to reduce the build-up of immunity in children and adults.

The second is the use of a deterministic mathematical model framework. Our reasons for doing so relate to the fairly predictable population dynamics of STH, where post perturbation the population bounces back to a pre-perturbation level in a monotonic manner. Our preliminary analyses of individual based stochastic models show that mean trajectories are in broad agreement with the deterministic results. Where they differ concerns the fact that a stochastic framework permits the generation of a frequency distribution of extinction times for a given set of parameter values with treatment coverage sufficient to break transmission. As such the certainty of the deterministic predictions is false – there will only be a certain probability of going extinct at the times documented in Table 3.

Our stochastic framework is also an individual-based approach and as such permits the exploration of more causes of heterogeneity, such as pre-disposition to heavy or light infection. Most importantly, they do permit exploring the consequences of relaxing our assumption of a random choice of individuals to treat at each round of treatment. It seems likely from studies of MDA for other helminths such as onchocerciasis and lymphatic filariasis [40, 41], that those not treated may be predisposed to this state of non-compliance by behavioural or social factors. This topic will be the subject of a future publication.

Mathematical models play an important role in infectious disease epidemiology, not just in prediction, but also in helping define what needs to be measured to facilitate a better understanding of observed pattern. One factor that emerges from our present analyses is the definition of what constitutes low, medium and high transmission settings. WHO defines these in terms of prevalence bands from epg measures for each species (and for schistosomes) [42]). Given the high variability of these counts due to poor diagnostic capabilities, epg measurements are poor, inaccurate indicators of transmission intensity. However, the value of faecal samples as a source for the accurate measurement of infection intensity may change in the future as new quantitative PCR methods become available.

The model validation analyses described in the results section is the first such attempt for models of helminth infection transmission dynamics (and response to control interventions). The results are encouraging and give confidence for the use of the model to provide the template for policy formulation for MDA control of STH. There is one discrepancy, however, concerning the prediction of reinfection in 5–10 year olds, where a much faster reinfection rate occurs than predicted. One implication of this feature is that *Ascaris* in children is probably more resilient to treatment than suggested by our current model results. This will be explored in a future publication, with model modifications to include age dependency in the negative binomial aggregation parameter, *k*, and a ‘who infects whom’ matrix that gives greater importance to children largely infecting each other. Studies based on whole parasite genome sequencing could help provide independent data on who infects whom in future molecular epidemiological studies. Excepting the anomaly for re-infection of children, the re-infection distribution is a reasonable fit. One conclusion from this is that the region studied was largely epidemiologically self-contained: no force of infection outside that generated by the parasites within the model was required achieve the observed bounce-back. Given that the original study was carried out in rural India in the early 80s, perhaps the epidemiological isolation is not surprising. With increasing human mobility, the movement of infectious worms and infectious material over long distances may become important, particularly for the re-introduction of parasites into a region where elimination has been achieved. More relevant to the understanding of current endemic patterns of infection are the regular movements within communities, e.g. children moving between school and village. Studies are currently under way which will collect data on these aspects of spatial heterogeneity.

## Conclusion

We return to the aims of this study; namely, to evaluate the potential impact of current WHO health policy aims for STH. Formerly, these assessments have been made on the basis of experience and expert opinion. However, with the increasing availability of relevant data and the mathematical tools to use it to inform models of parasite dynamics and response to treatment, this process should be put on a more scientific footing. When the World Health Assembly announces broad goals for the future control of NTDs, it is highly desirable that these aims are accompanied by calculations on what is precisely needed to achieve them, year by year of the time frame defined for the achievement of the stated goals. We hope the current analyses reported in this paper highlight the value of doing so.

## Additional file

Additional file 1:
**Details of transmission model and fitting results.** (PDF 640 KB)
