# Supplementary material for: What impact will the achievement of the current World Health Organisation targets for anthelmintic treatment coverage in children have on the intensity of soil transmitted helminth infections?
Source: Parasit Vectors. 2015 Oct 22;8:551. doi: 10.1186/s13071-015-1135-4 (PMC4618937; doi:10.1186/s13071-015-1135-4)
Supplement: Additional file 1: — Details of transmission model and fitting results. (PDF 640 KB) [file 13071_2015_1135_MOESM1_ESM.pdf]

Supplementary information

The Model

The fundamental model used to describe the mean worm burden of individuals of a given age and the quantity of infectious eggs in the environment was developed from the founding work of Anderson and May [1]. The current version of the model is described in detail in [2, 3]. Briefly, the model is an ODE model describing the evolution of mean female worm burden as a function of age, M(a,t)

$$\frac{\partial M(a,t)}{\partial t}+\frac{\partial M(a,t)}{\partial a}=L\beta(a)-\sigma M(a,t)$$

where L is the concentration of infectious material in the environment. The model describes the evolution of the female worm burden and assumes they are distributed according to an underlying negative binomial distribution. The dynamics of infectious material is governed by

$$\frac{dL}{dt}=\psi\lambda\int_{a=0}^{\infty}M(a)f(M(a),z,k)\rho(a)P(a)da-\mu_2 L$$

where *P(a)* is the normalised age distribution for the population. The function $f(.)$ describes the production of fertile infectious material and is the product of a term representing the dampening effect of density dependent fecundity at higher worm burdens [first term] and the catalytic effect of the presence of male worms on sexual reproduction at very low worm burdens [second term] [4]

$$f(M,z,k)=[1+(1-z)M/k]^{-(k+1)}\left(1-\left[\frac{1+M(1-z)/k}{1+M(2-z)/k}\right]^{k+1}\right)$$

where $z=e^{-\gamma}$ representing the strength of density-dependent fecundity and *k* is the negative binomial aggregation parameter, as discussed in the main text. The parameter ψ characterises the flow of infectious material into the environment. This parameter and the absolute magnitude of β and ρ are subsumed into the definition of the basic reproduction number, R_0, that measures the intensity of the transmission cycle.

$$R_0=\frac{z\lambda\psi}{\mu_2}\int_{a=0}^{\infty}\rho(a)P(a)\int_{x=0}^{a}\beta(x)e^{-\sigma(a-x)}dxda$$

Assuming a 1:1 sex ratio in worms, the total worm burden is given by $2M(a,t)$.

Treatment is modelled without systematic non-compliance. Coverage in an age group is taken as the probability that an individual in that age group will receive treatment. Hence the drop in mean worm burden from treatment is the product of the coverage and drug efficacy (which can be understood

as the probability of a single treatment killing a worm within the host). We have equated this with the faecal egg count reduction (FECR) parameter, although it has been observed that FECR has a non-linear relationship with egg count, making our assumption slightly pessimistic [5].

Credible intervals for parameters

Table S2 shows the credible intervals associated with the parameter estimates shown in Table 1 in the main text. Hookworm parameter estimates were based on only a few age-averaged data points (See Fig 2 in the main text) and hence the uncertainties in those estimates are not representative and are omitted.

Table S1 Parameter values estimated using maximum likelihood methods and used in the numerical evaluations of model predictions for each parasite. Figures in brackets represent the 95% credible intervals for the around the maximum likelihood estimators. The age intensity profiles from the cited sources based on worm expulsion were used to derive estimate of R_0, β_i and k. The values of ɣ were derived from egg per gram of faeces and worm expulsion counts [transmission age groups: Ascaris and hookworm: 0-2;2-5;5-15;15+; Trichuris: 0-2;2-7;7-12;12+].

**Parameter**	***Ascaris***	***Trichuris***	**Hookworm**	**Source**
Basic Reproductive number, R_0	2.12 [1.7,3.2]	1.72 [1.5,3.4]	2.34	Fitted
Negative binomial clumping parameter, k	0.90 [0.75,1.12]	0.38 [0.29,0.48]	0.35	Fitted
Density dependence fecundity parameter, ɣ	0.07 [0.05,0.1]	0.0035 [0,0.006]	0.08	Fitted
Age-specific transmission parameter, β_i for age group i's contact with infectious reservoir. Relative egg contribution by age, ρ_i, proportional to β_i.	0.22 [0.12,0.55],<br>1.88 [1.1,3.2],<br>1 [set],<br>0.53 [0.36,0.8]	0.5 [0.4,2.3],<br>2.13 [2,9.4],<br>1 [set],<br>0.28 [0.24,1.9]	0.03,<br>0.09,<br>1,<br>2.5	Fitted

Threshold sensitivity

Given the wide ranging and long-term morbidity factors associated with STH burden, any proxy measure will have considerable uncertainty associated with it. In their original study, Bundy et al recognise the approximate nature of these thresholds and define two sets; a lower set based as far as possible on empirical observations of worm numbers and associated morbidity factors and a more conservative set, with values twice as high, to act as a lower bound on morbidity estimates [6]. In Fig S1, we compare the impact of the coverage trend on the percentage of the population with high worm burdens in Ascaris for the lower thresholds (Fig S1 A) and for the lower bound thresholds (Fig S1 B). For high mean worm burdens, the higher thresholds lead to proportionately lower

percentages with high burdens, but for low mean burdens, such as is present in the adult population and in the overall mean, the effect is exaggerated. In the adult population, high burden drops from 5% to effectively zero with the change in thresholds. It's clear that in using these type of proxy indicators of morbidity, both the absolute value and its distribution among age groups can change radically under different assumptions. As such, results based on such proxies should be treated with appropriate caution.

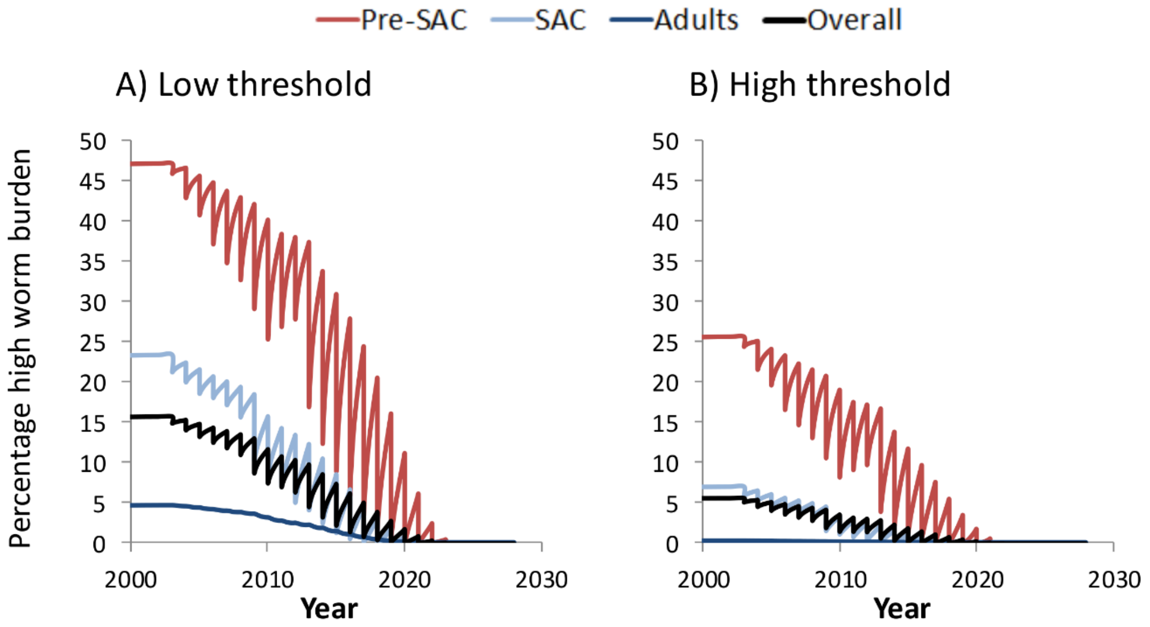

Fig S1: Evolution of percentage high worm burden in Ascaris under treatment regime shown in Fig 1 (main text). A) Low age-dependent worm threshold: 10 [0-4 years], 15 [5-9 years], 20 [10+ years]. B) 20 [0-4 years], 30 [5-9 years], 40 [10+ years].(From [6]).

References

1. Anderson RM, May RM: **Herd immunity to helminth infection and implications for parasite control**. *Nature* 1985, **315**(6019):493-496.
2. Truscott JE, Hollingsworth TD, Brooker SJ, Anderson RM: **Can chemotherapy alone eliminate the transmission of soil transmitted helminths?** *Parasites & vectors* 2014, **7**(1):266.
3. Truscott JE, Turner HC, Farrell SH, Anderson RM: **Soil Transmitted Helminths: mathematical models of transmission, the impact of mass drug administration and transmission elimination criteria**. In: *Mathematical Models for Neglected Tropical Diseases: Essential Tools for Control and Elimination. Volume B*, edn.; 2015.
4. May RM: **Togetherness among Schistosomes: its effects on the dynamics of the infection**. *Mathematical biosciences* 1977, **35**:301-343.
5. Levecke B, Anderson RM, Berkvens D, Charlier J, Devleesschauwer B, Speybroeck N, Vercruysse J, Van Aelst S: **Mathematical inference on helminth egg counts in stool and its applications in mass drug administration programmes to control soil-transmitted helminthiasis in public health**. *Advances in parasitology* 2015, **87**:193-247.

6. Bundy DAP, Chan MS, Medley GF, Savioli L (eds.): **Intestinal nematode infections**: World Health Organization; 2004.
